# MicroRNA-141-3p reduces pulmonary hypoxia/reoxygenation injury through suppression of Beclin-1-dependent autophagy

**DOI:** 10.18632/aging.205430

**Published:** 2024-01-22

**Authors:** Yanping Zhan, Lei Li, Chen Guo, Yang Zhang, Lili Zhao, Zhe Tao, Hua Zhang, Shibiao Chen

**Affiliations:** 1 Department of Anesthesiology, The First Affiliated Hospital of Nanchang University, Nanchang 330006, P.R. China; 2 Jiangxi Maternal and Child Health Hospital, Nanchang 330006, P.R. China; 3 Nanchang University, Nanchang 330006, P.R. China

**Keywords:** autophagy, hypoxia/reoxygenation injury, microRNA-141-3p, SIRT1, Beclin-1

## Abstract

Alterations in autophagy are involved in pulmonary hypoxia/reoxygenation (H/R)-induced injury. Here, we intended to explain the function of microRNA-141-3p (miR-141-3p) in regulating autophagy under the H/R condition. Rat pulmonary microvascular endothelial cells (PMVECs) were applied for H/R cell model establishment, followed by tracing of autophagy formation. SIRT1 plays a critical role in controlling the lifespan of yeast, flies, and mice. Interaction between SIRT1 and Beclin-1, an indicator protein for autophagy, and between miR-141-3p and SIRT1 was assayed with their roles in PMVEC injury. Autophagy of PMVECs was activated after hypoxia treatment and further activated after H/R treatment. The binding of miR-141-3p and SIRT1 was verified. In H/R-treated PMVECs, the binding of miR-141-3p and SIRT1 was reduced. Furthermore, SIRT1 acted as a deacetylase to stabilize the Beclin-1 protein, promoting autophagy and PMVEC injury. H/R rat models were established, and *in vivo*, experiments further confirmed that miR-141-3p regulated autophagy and lung injury in H/R rats through SIRT1/Beclin-1 axis. The current study highlighted that reduced miR-141-3p in H/R-treated PMVECs promoted deacetylation of Beclin-1 by SIRT1, thus causing PMVEC injury.

## INTRODUCTION

Ischaemia following reperfusion can be seen frequently in many medical conditions, including significant surgical processes and organ transplantation, often causing fatal consequences, such as tissue injury [1]. Lung ischemia-reperfusion (I/R) injury features interstitial edema, infiltration of inflammatory cells, and disruption of respiratory membranes accompanied by increased morbidity and mortality [2]. Moreover, as a complex inflammatory response, I/R injury entails endothelial and epithelial injury/dysfunction [3]. It has been reported that when endothelial cells are exposed to hypoxia and reoxygenation (H/R) stimulation, endothelial cell-vascular interactions are disturbed, permeability is altered, proliferation of peripheral smooth muscle cells is disrupted, and apoptosis is induced [4, 5]. H/R-induced apoptosis of endothelial cells can lead to pulmonary arterial hypertension, pulmonary edema and fibrosis [4, 6, 7]. Thus, a better understanding of the effects of H/R on PMVECs may help to develop therapeutic strategies that increase graft survival and reduce the risk of these diseases.

It has been reported that autophagy is involved in pulmonary I/R injury [2]. Expression of the Beclin-1 protein mediates the upregulation of autophagy and reflects the level of autophagy, which is significantly elevated during reperfusion [8]. Beclin-1 is a mammalian protein homologous to the yeast autophagy-associated protein Apg6/Vps30, which has a powerful effect on regulating autophagy and cell death [9, 10].

Importantly, microRNAs (miRNAs) have emerged as critical regulators of I/R injury and have been implicated in the pathogenesis of organ rejection [11]. Many miRNAs, including miR-1 and miR-93, are associated with H/R injury [12, 13]. The relationship between H/R and miR-141-3p is largely unknown and controversial. It has been reported that H/R significantly reduces the expression of miR-141-3p, and miR-141-3p mimics significantly reduce H/R-induced apoptosis in cardiomyocytes [14]. However, it is reported that under hypoxic conditions, the inhibition of miR-141-3p activates the PI3K/AKT signaling pathway and reduces rat cardiomyocyte apoptosis [15]. This aroused our interest to investigate the role and mechanism of miR-141-3p in pulmonary H/R injury in depth.

Previous studies have indicated that miR-141-3p may interact with sirtuin 1 (SIRT1) [16, 17]. SIRT1 involves various cells’ metabolism, inflammation, and cell cycle/apoptosis [18]. Notably, a recent study has reported that miR-34a/SIRT1 regulates autophagy, activates autophagy and inhibits apoptosis [19].

Based on the above research, we speculated that miR-141-3p/SIRT1/Beclin-1 axis may involve autophagy and induce H/R injury of PMVECs. In the present study, we isolated PMVECs from rats and exposed them to normoxia, hypoxia and H/R, followed by autophagy detection and observation of cell injury. PMVECs were treated with established plasmids or reagents to explore further the mechanism to determine the potential relationship among miR-141-3p, SIRT1 and Beclin-1 during H/R injury.

## RESULTS

### 3-MA treatment could effectively attenuate H/R-induced PMVEC injury

It has been previously demonstrated that autophagy is involved in donor lung I/R injury, and inhibiting autophagy can prevent the injury for lung transplantation [20], but the mechanism of how autophagy functions in H/R requires further investigation. Herein, we isolated rat PMVECs and cultured them under hypoxia and H/R. The expression of marker CD31 was detected by immunofluorescence (Supplementary Figure 1A), and the results showed that PMVECs were successfully isolated.

Firstly, we constructed a cell model of PMVECs as previously described [20, 21] and detected the expression of two hypoxia marker genes, HIF-1α and PDK1 [22], to ensure that cells did respond to hypoxia treatment. The results of RT-PCR showed that the levels of HIF-1α and PDK1 gradually elevated with the increase of hypoxia time (Supplementary Figure 1B). Meanwhile, flow cytometry results revealed that hypoxia treatment significantly increased cell apoptosis, and the apoptosis was more evident with the increase in hypoxia time (Supplementary Figure 2A). All these results indicated the successful model construction. Western blot results demonstrated that LC3-II/LC3-I and Beclin-1 expression increased significantly with the increase of hypoxia time (Supplementary Figure 2B).

Moreover, the fluorescence detection results also showed that the number of autophagosomes and autophagolysosomes increased gradually with the increase of hypoxia time (Supplementary Figure 2C). These results suggested that the autophagy level of H/R-induced cells increased significantly and gradually in a time-dependent hypoxia. In combination with the relevant results of the existing literature [20, 21], we selected 8 h hypoxia induction time for subsequent study.

**Figure 1:**
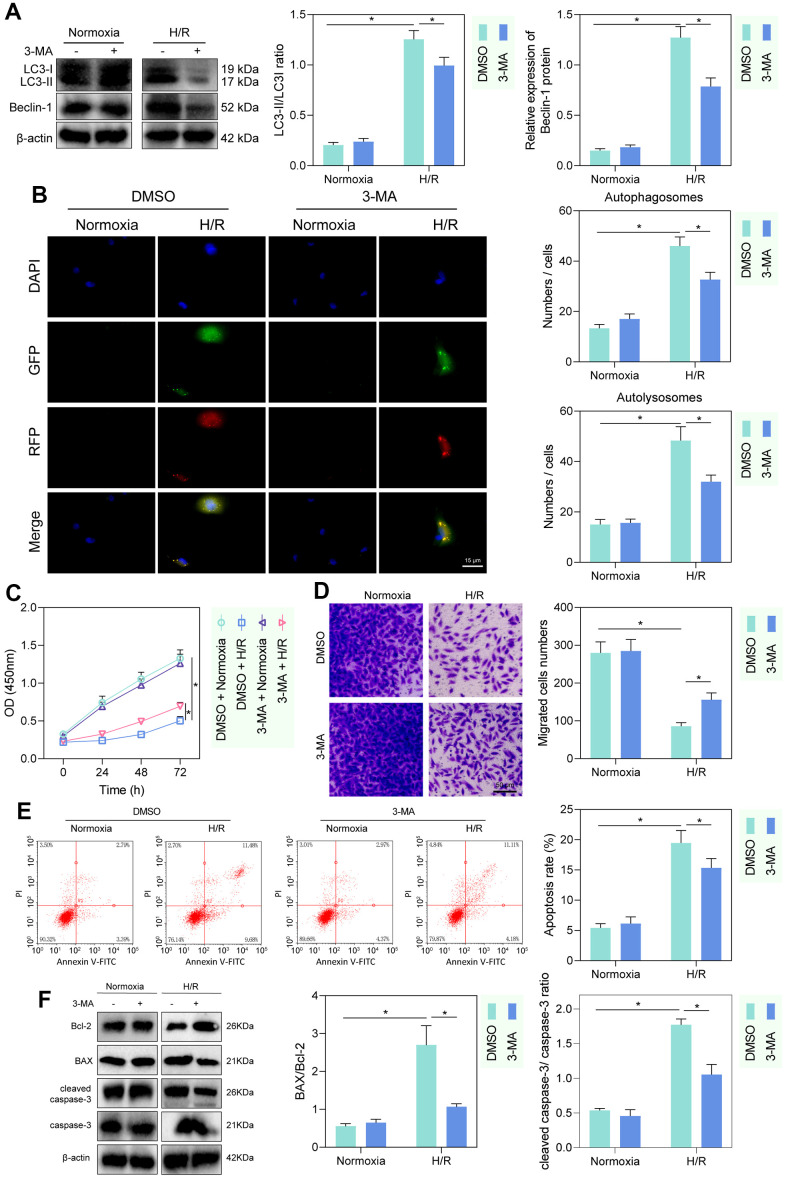
**H/R enhanced PMVEC injury via autophagy.** (**A**) Western blot showing the expression of LC3-I/LC3-II and Beclin-1 under treatment of 3-MA, normoxia, hypoxia or H/R. (**B**) The mRFP-GFP-LC3 dual fluorescence system was used to trace autophagy formation under treatment of 3-MA, normoxia, hypoxia or H/R. (**C**) CCK-8 assay showing changes in proliferation of cells treated with 3-MA or under normoxia, hypoxia or H/R. (**D**) Transwell assay showing PMVEC migration (magnification, 200 ×; scale bar = 50 μm) under treatment of 3-MA, normoxia, hypoxia or H/R. (**E**) Flow cytometry showing apoptosis under treatment of 3-MA, normoxia, hypoxia or H/R. (**F**) Western blot showing Bcl-2 and Bax, cleaved caspase-3 and caspase-3 expression under treatment of 3-MA, normoxia, hypoxia or H/R. Measurement data are expressed as mean ± standard deviation. Differences between 2 groups of data were compared using an unpaired *t*-test. Two-way ANOVA was used for data comparison at different time points. * *p* < 0.05. The cell experiments were repeated three times.

The autophagy inhibitor 3-MA was applied to inhibit cellular autophagy. Western blot detection showed that LC3-II/LC3-I and Beclin expression was increased in PMVECs under H/R treatment relative to those under hypoxia. 3-MA intervention hardly altered the expression of LC3-II/LC3-I and Beclin 1 in normoxia-treated PMVECs, but significantly reduced the expression of LC3-II/LC3-I and Beclin-1 in H/R-treated PMVECs (Figure 1A). Autophagy formation was further traced using the mRFP-GFP-LC3 dual fluorescence system, and the results observed that LC3-II was diffusely under-expressed in normoxia-treated PMVECs with weak expression intensity. In H/R-treated PMVECs, LC3-II expression was enhanced with increased autophagosomes and autophagolysosomes. In the presence of 3-MA, the fluorescence intensity effectively decreased, and the formation of autophagosomes reduced as well (Figure 1B).

To detect the injury of hypoxia and H/R treatment on PMVECs, we further detected cell proliferation and migration by CCK-8 and Transwell assays. The proliferation and migration of PMVECs were shown to be inhibited by H/R. Besides, administration of 3-MA promoted proliferation and migration in H/R-treated PMVECs (Figure 1C, 1D). In addition, apoptosis of PMVECs increased under H/R treatment and reduced after the further 3-MA intervention, suggesting that autophagy-mediated apoptosis in H/R treated PMVECs (Figure 1E). Moreover, under H/R, Bcl-2 expression decreased, and Bax expression, as well as cleaved caspase-3/caspase-3 ratio, increased more remarkably, while after further 3-MA intervention, Bcl-2 was elevated and Bax as well as cleaved caspase-3/caspase-3 ratio was decreased, indicating that autophagy promoted apoptosis under H/R (Figure 1F). The above results indicated that autophagy could contribute to injury in H/R-treated PMVECs to some extent.

### SIRT1 promoted H/R-induced PMVEC autophagy and injury by increasing deacetylation of Beclin-1

**Figure 2:**
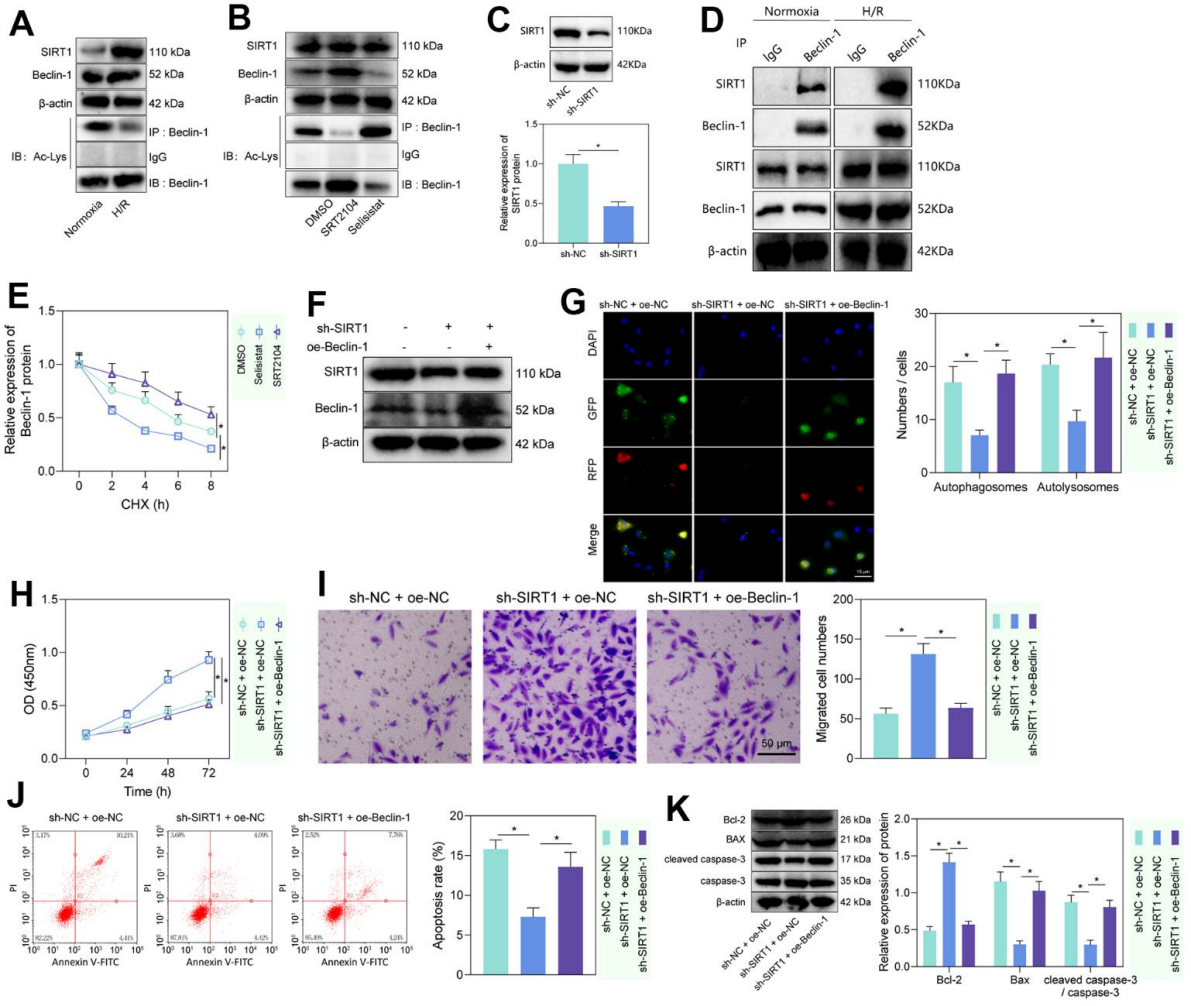
**SIRT1 deacetylated Beclin-1 to enhance autophagy and injury of H/R-treated PMVECs.** (**A**) Western blot showing the expression of SIRT1, Beclin-1, and Beclin-1-Ac under normoxia or H/R. (**B**) Western blot showing expression of Beclin-1 and Beclin-1-Ac under treatment with SRT2104 or Selisistat. (**C**) Western blot showing SIRT1 expression in PMVECs transfected with sh-SIRT1. (**D**) Detection of the interaction of SIRT1 with Beclin-1 in PMVECs with oe-SIRT1 under normoxia or H/R condition by Co-IP assay. (**E**) Beclin-1 protein stability analysis based on CHX treatment. (**F**) Western blot showing SIRT1, Beclin-1, and Beclin-1-Ac expression in H/R-treated PMVECs with oe-Beclin-1 and sh-SIRT1. (**G**) The mRFP-GFP-LC3 dual fluorescence system was used to trace autophagy formation after transfection of sh-SIRT1 or oe-Beclin-1 (magnification, 400 ×; scale bar = 25 μm). (**H**) Detection of changes in cell proliferation by CCK-8 assay after transfection of sh-SIRT1 or oe-Beclin-1. (**I**) Detection of PMVEC migration by Transwell assay (magnification, 200 ×; scale bar = 50 μm) after transfection of sh-SIRT1 or oe-Beclin-1. (**J**) Detection of apoptosis by flow cytometry after transfection of sh-SIRT1 or oe-Beclin-1. (**K**) Detection of Bax, Bcl-2, cleaved caspase-3 and caspase-3 expression by Western blot after transfection of sh-SIRT1 or oe-Beclin-1. Measurement data are expressed as mean ± standard deviation. Differences between 2 groups of data were compared using an unpaired t-test. Changes between multiple groups were compared using one-way ANOVA and Tukey’s multiple comparison test. Two-way ANOVA was used for data comparison at different time points. * *p* < 0.05. The cell experiments were repeated three times.

The histone deacetylase SIRT1 has been reported to promote cellular autophagy and apoptosis by deacetylating Beclin-1 [23, 24]. To test whether this regulation existed in rat PMVECs, we first performed Western blot and IP-Western blot and found increased SIRT1 and Beclin-1 and decreased Beclin-1-Ac in H/R-treated PMVECs (Figure 2A). Further treatment of PMVECs with the SIRT1 agonist SRT2104 increased Beclin-1 expression and a decrease in Beclin-1-Ac expression, while SIRT1 antagonist Selisistat had the opposite effect, suggesting that SIRT1 significantly decreased Beclin-1 acetylation and increased its protein expression (Figure 2B). The results from Western blot analysis revealed a significant decrease in the protein levels of SIRT1 in the sh-SIRT1 group compared to the sh-NC group (Figure 2C). Based on Co-IP assay results, there was interaction between endogenous SIRT1 and Beclin-1 either in normoxic or H/R state; however, in normoxic state, the interaction was weaker due to the lower protein expression of between SIRT1 and Beclin-1 in PMVECs (Figure 2D). Because acetylation can regulate protein stability [25], a CHX-based protein stability analysis was further carried out. SRT2014 enhanced the stability of Beclin-1 protein, while Selisistat reduced it. These results demonstrated that SIRT1 promoted the stability and expression of Beclin-1 protein through its effect of deacetylation (Figure 2E and Supplementary Figure 3A).

To test whether SIRT1 regulates PMVEC injury via Beclin-1, Beclin-1 overexpression efficiency was tested, three SIRT1 silencing sequences were constructed, and sh-SIRT1-1 with the highest silencing efficiency was selected for the next experiment (Supplementary Figure 3B). Then, we found that sh-SIRT1 treatment reduced SIRT1 and Beclin-1 expression, while further overexpression of Beclin-1 caused no alteration in SIRT1 expression but elevated Beclin-1 expression (Figure 2F). Additionally, silencing SIRT1 suppressed autophagy, manifested by reduced autophagosomes and autophagolysosomes, while further overexpression of Beclin-1 could reverse the effects (Figure 2G). The expression of LC3-II/LC3-I detected by Western blot revealed that silencing SIRT1 could significantly inhibit the expression of LC3-II/LC3-I, while overexpression of Beclin-1 reversed the effect caused by SIRT1 silencing alone (Supplementary Figure 3C). Our results from CCK-8 and Transwell assays showed that silencing SIRT1 facilitated the proliferation and migration of PMVECs, which was reversed by overexpression of Beclin-1 (Figure 2H, 2I). Moreover, the results of flow cytometry and Western blot showed together that silencing SIRT1 suppressed apoptosis, as evidenced by reduced Bax expression, diminished cleaved caspase-3/caspase-3 ratio and elevated Bcl-2 expression, while overexpression of Beclin-1 reversed the effects (Figure 2J, 2K). The above results indicated that SIRT1 facilitated the deacetylation of Beclin-1, thereby inducing PMVEC autophagy and injury.

### H/R promoted the expression of SIRT1 in PMVECs by inhibiting miR-141-3p

**Figure 3:**
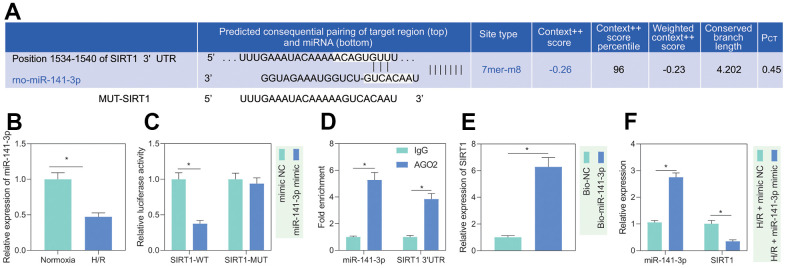
**miR-141-3p targeted SIRT1 in H/R-treated PMVECs.** (**A**) miR-141-3p had a binding site in the 3’UTR of SIRT1, predicted by the TargetScan website. (**B**) miR-141-3p expression detected by RT-qPCR under normoxia or H/R. (**C**) Binding of miR-141-3p and SIRT1 confirmed by dual luciferase assays. (**D**) Binding of miR-141-3p and SIRT1 detected by RIP. (**E**) Binding of miR-141-3p and SIRT1 detected by RNA pull-down. (**F**) Expression of miR-141-3p and SIRT1 detected by RT-qPCR in H/R-treated PMVECs with miR-141-3p mimic under normoxia or H/R. Measurement data are expressed as mean ± standard deviation. Differences between 2 groups of data were compared using an unpaired t-test. Data among multiple groups were compared using one-way ANOVA and Tukey’s multiple comparison test. * *p* < 0.05. The cell experiments were repeated three times.

It is known that miR-141-3p is significantly reduced in H/R-treated cardiomyocytes [14]. Additionally, miR-141-3p has a binding site at the 3’UTR of SIRT1, predicted by the TargetScan website (Figure 3A). Based on the evidence, we speculated that miR-141-3p targeted SIRT1 in PMVECs treated with H/R. RT-qPCR results showed that miR-141-3p expression was significantly reduced after H/R treatment (Figure 3B). Besides, dual luciferase assay revealed that miR-141-3p mimic significantly inhibited luciferase activity in cells with SIRT1-WT but had no significant effect on that in cells with SIRT1-MUT, indicating that miR-141-3p can bind SIRT1 3’UTR (Figure 3C), which was consistent with the results from RIP (Figure 3D) and RNA pull-down assay (Figure 3E). Further, reduced miR-141-3p and increased SIRT1 under H/R were rescued by miR-141-3p mimic (Figure 3F), suggesting that H/R treatment promoted SIRT1 expression in PMVECs by inhibiting miR-141-3p.

### H/R promoted PMVEC autophagy and injury by inhibiting miR-141-3p

**Figure 4:**
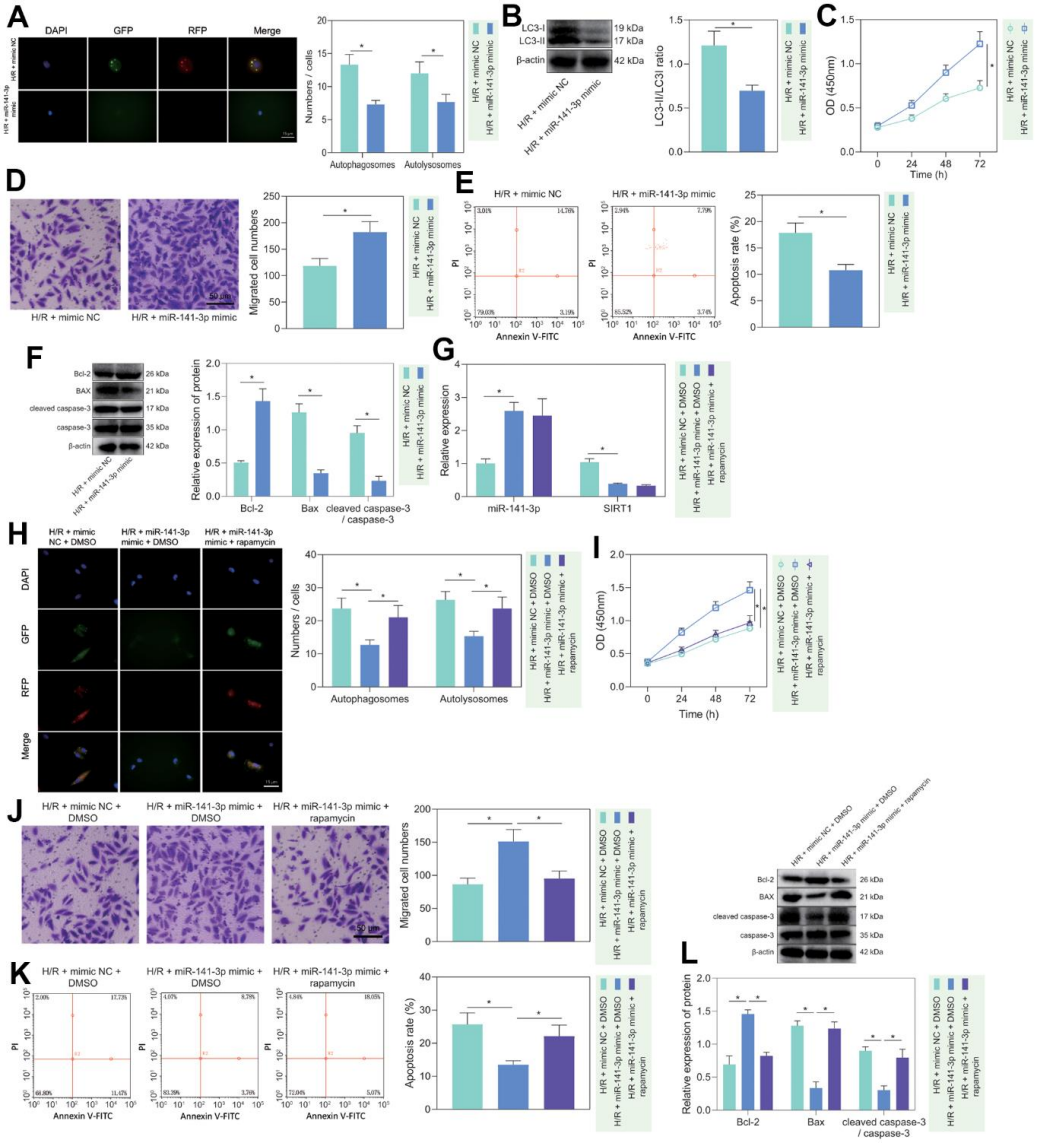
**miR-141-3p repressed H/R-induced autophagy and alleviated PMVEC injury.** (**A**) The mRFP-GFP-LC3 dual fluorescence system was used to trace autophagy formation in PMVECs treated with H/R and miR-141-3p mimic. (**B**) The expression of LC3-I/LC3-II in H/R-exposed PMVECs treated with miR-141-3p mimic detected by Western blot. (**C**) The proliferation of H/R-exposed PMVECs treated with miR-141-3p mimic detected by CCK-8 assay under normoxia or H/R. (**D**) The migration of H/R-exposed PMVECs treated with miR-141-3p mimic detected by Transwell assay. (**E**) The apoptosis of H/R-exposed PMVECs treated with miR-141-3p mimic detected by flow cytometry. (**F**) The expression of Bax, Bcl-2, cleaved caspase-3 and caspase-3 in H/R-exposed PMVECs treated with miR-141-3p mimic detected by Western blot. (**G**) The expression of miR-141-3p and SIRT1 detected by RT-qPCR in H/R-exposed PMVECs treated with miR-141-3p mimic or combined with Rapamycin. (**H**) mRFP-GFP-LC3 dual fluorescence system was used to trace autophagy formation in H/R-exposed PMVECs treated with miR-141-3p mimic alone or combined with Rapamycin. (**I**) The proliferation of H/R-exposed PMVECs treated with miR-141-3p mimic alone or combined with Rapamycin detected by CCK-8 assay. (**J**) The migration of H/R-exposed PMVECs treated with miR-141-3p mimic alone or combined with Rapamycin detected by Transwell assay. (**K**) The apoptosis of H/R-exposed PMVECs treated with miR-141-3p mimic or combined with Rapamycin detected by flow cytometry. (**L**) The expression of Bax, Bcl-2, cleaved caspase-3 and caspase-3 in H/R-exposed PMVECs treated with miR-141-3p mimic alone or combined with Rapamycin detected by Western blot. Measurement data are expressed as mean ± standard deviation. Changes between multiple groups were compared using one-way ANOVA and Tukey’s multiple comparison test. Two-way ANOVA was used for data comparison at different time points. * *p* < 0.05. The cell experiments were repeated three times.

To investigate the regulatory role of miR-141-3p in H/R-induced injury, we treated miR-141-3p mimic to H/R-treated PMVECs and found that miR-141-3p mimic decreased the fluorescence intensity in the H/R-exposed PMVECs, and the autophagosomes and autophagolysosomes were decreased (Figure 4A). Western blot analysis revealed that the expression of LC3-II/LC3-I was decreased in H/R-exposed PMVECs after treatment with miR-141-3p mimic (Figure 4B). Moreover, functional assays demonstrated that cell proliferation (Figure 4C) and migration (Figure 4D) were enhanced, and apoptosis was suppressed (Figure 4E). Based on Western blot results (Figure 4F), miR-141-3p mimic resulted in declines in the expression of Bax and cleaved caspase-3/caspase-3 ratio and an increase in Bcl-2 expression in H/R-exposed PMVECs. The above results indicated that miR-141-3p inhibited H/R-induced PMVEC autophagy and injury.

To further clarify whether miR-141-3p mediated H/R-induced injury in PMVECs through inhibition of autophagy, we overexpressed miR-141-3p in H/R-treated PMVECs followed by Rapamycin treatment. The results of RT-qPCR depicted that miR-141-3p mimic effectively elevated miR-141-3p expression but reduced SIRT1 expression, while Rapamycin treatment exerted no alteration (Figure 4G). Besides, overexpression of miR-141-3p led to reduced Beclin-1 and LC3-II/LC3-I expression, while application of Rapamycin restored all of the alteration induced by miR-141-3p mimic (Supplementary Figure 3D). Moreover, the mRFP-GFP-LC3 dual fluorescence system was used to trace autophagy formation. The results showed that miR-141-3p mimic suppressed the fluorescence intensity and reduced autophagosomes and autophagolysosomes, while the addition of Rapamycin treatment reversed the effects (Figure 4H). miR-141-3p mimic significantly promoted cell proliferation (Figure 4I) and migration (Figure 4J) but suppressed apoptosis (Figure 4K), while the addition of Rapamycin treatment reversed the effects (Figure 4I–4K). Western blot results (Figure 4L) found that overexpression of miR-141-3p contributed to declines in the expression of Bax and cleaved caspase-3/caspase-3 ratio and an increase in Bcl-2 expression in H/R-exposed PMVECs, and all these effects could be negated by Rapamycin treatment. The above results indicated that miR-141-3p could inhibit H/R-induced PMVEC autophagy and cell injury.

### miR-141-3p inhibited H/R-induced autophagy and injury in PMVECs via SIRT1/Beclin-1 axis

**Figure 5:**
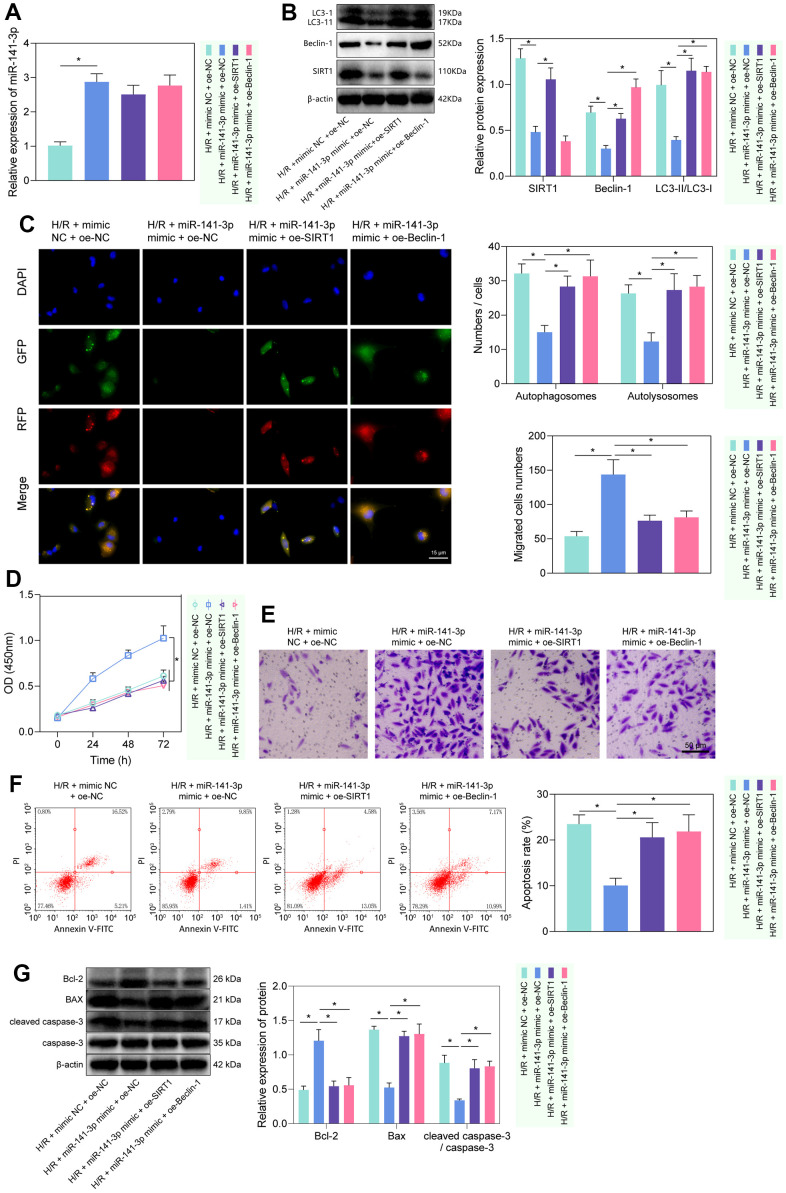
**miR-141-3p mimic suppressed PMVEC autophagy and injury via the SIRT1/Beclin-1 axis.** (**A**) The expression of miR-141-3p detected by RT-qPCR in H/R-exposed PMVECs treated with miR-141-3p mimic alone or combined with oe-SIRT1 or oe-Beclin-1. (**B**) The expression of SIRT1, Beclin-1, LC3-I/LC3-II detected by Western blot in H/R-exposed PMVECs treated with miR-141-3p mimic alone or combined with oe-SIRT1 or oe-Beclin-1. (**C**) mRFP-GFP-LC3 dual fluorescence system was used to trace autophagy formation in H/R-exposed PMVECs treated with miR-141-3p mimic alone or combined with oe-SIRT1 or oe-Beclin-1. (**D**) The proliferation of H/R-exposed PMVECs treated with miR-141-3p mimic alone or combined with oe-SIRT1 or oe-Beclin-1 detected by CCK-8 assay. (**E**) The migration of H/R-exposed PMVECs treated with miR-141-3p mimic alone or combined with oe-SIRT1 or oe-Beclin-1 detected by Transwell assay. (**F**) The apoptosis of H/R-exposed PMVECs treated with miR-141-3p mimic alone or combined with oe-SIRT1 or oe-Beclin-1 detected by flow cytometry. (**G**) The expression of Bax, Bcl-2, cleaved caspase-3 and caspase-3 in H/R-exposed PMVECs treated with miR-141-3p mimic alone or combined with oe-SIRT1 or oe-Beclin-1 detected by Western blot. Measurement data are expressed as mean ± standard deviation. Changes between multiple groups were compared using one-way ANOVA and Tukey’s multiple comparison test. Two-way ANOVA was used for data comparison at different time points. * *p* < 0.05. The cell experiments were repeated three times.

We then investigated the regulatory role of miR-141-3p in H/R-induced injury via the SIRT1/Beclin-1 axis. As reflected by RT-qPCR and Western blot analysis, miR-141-3p mimic elevated the expression of miR-141-3p, and reduced the expression of SIRT1, Beclin-1, and LC3-II/LC3-I, while further treatment with oe-SIRT1 or oe-Beclin-1 rescued the effects (Figure 5A, 5B). Then, we found that miR-141-3p mimic inhibited autophagy, manifested by reduced autophagosomes and autophagolysosomes, while further overexpression of SIRT1 or Beclin-1 could reverse the effects (Figure 5C). The results of CCK-8 and Transwell assays showed that miR-141-3p mimic promoted the proliferation and migration of PMVECs, which was reversed by overexpressed SIRT1 or Beclin-1 (Figure 5D, 5E). Additionally, flow cytometry results revealed that overexpressed miR-141-3p suppressed apoptosis, while oe-SIRT1 or oe-Beclin-1 reversed the effects (Figure 5F). Western blot results (Figure 5G) found that overexpression of miR-141-3p decreased the expression of Bax and cleaved caspase-3/caspase-3 ratio while increasing Bcl-2 expression in H/R-exposed PMVECs, and all these effects could be negated by overexpressed SIRT1 or Beclin-1. The above results indicated that miR-141-3p could suppress H/R-induced autophagy and injury of PMVECs through regulation of the SIRT1/Beclin-1 axis [21, 26].

### miR-141-3p regulated lung injury in H/R rats via SIRT1/Beclin-1 axis

**Figure 6:**
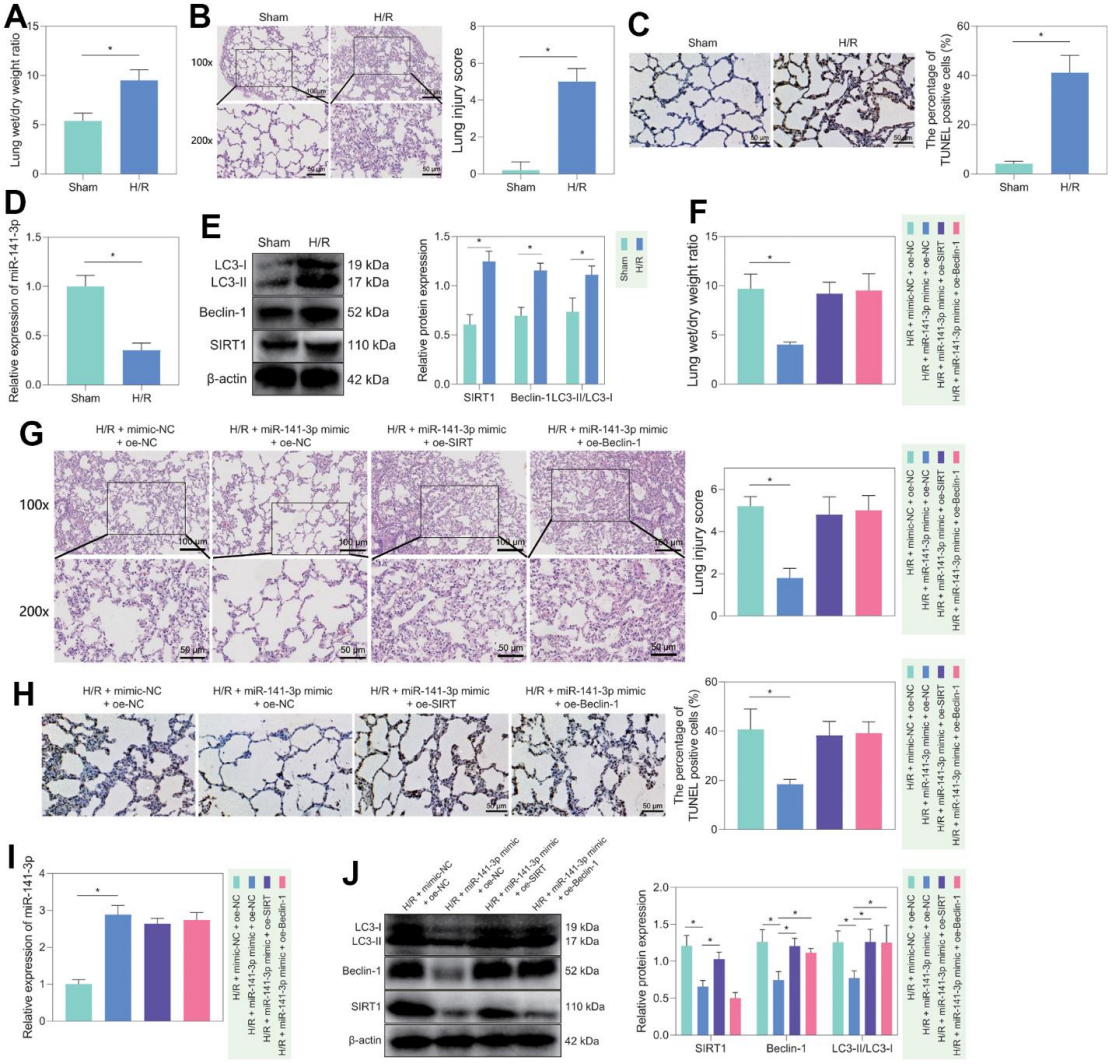
**H/R rat model construction and *in vivo* validation of miR-141-3p/SIRT1/Beclin-1 axis regulatory function.** (**A**) Dry/wet weight ratio of lung tissue of sham-operated rats and H/R rats. (**B**) HE staining for detecting the lung tissue injury of sham-operated and H/R rats. (**C**) TUNEL staining for detecting the apoptosis of lung tissue in sham-operated and H/R rats. (**D**) The level of miR-141-3p in lung tissue of sham-operated rats and H/R rats detected by RT-qPCR. (**E**) Western blot to detect SIRT1, Beclin-1, LC3-II/LC3-I levels in lung tissue of sham-operated and H/R rats. (**F**) Dry/wet weight ratio of lung tissue of H/R rats in response to miR-141-3p mimic alone or combined with oe-SIRT1 or oe-Beclin-1. (**G**) HE staining to detect the lung tissue injury of H/R rats in response to miR-141-3p mimic alone or combined with oe-SIRT1 or oe-Beclin-1. (**H**) TUNEL staining to detect the apoptosis of lung tissue in H/R rats in response to miR-141-3p mimic alone or combined with oe-SIRT1 or oe-Beclin-1. (**I**) The level of miR-141-3p in lung tissue of H/R rats in response to miR-141-3p mimic alone or combined with oe-SIRT1 or oe-Beclin-1 detected by RT-qPCR. (**J**) Western blot to detect the levels of SIRT1, Beclin-1 and LC3-II/LC3-I in lung tissue of H/R rats in response to miR-141-3p mimic alone or combined with oe-SIRT1 or oe-Beclin-1. n = 5. Measurement data are expressed as mean ± standard deviation. Changes between multiple groups were compared using one-way ANOVA and Tukey’s multiple comparison test. Two-way ANOVA was used for data comparison at different time points. * *p* < 0.05.

Furthermore, we verified the regulatory role of miR-141-3p/SIRT1/Beclin-1 axis in lung tissue *in vivo*. We first constructed an H/R rat model and weighed the rat lung tissues. We found that compared with sham-operated rats, H/R rats had prominent pulmonary edema (Figure 6A). In addition, HE staining displayed that the alveolar structure of sham-operated rats was orderly, without hyperemia, neutrophil infiltration or interstitial edema, while the H/R rats had disordered alveolar structure and damaged integrity of the alveolar wall, accompanied by thickening and edema of the alveolar wall, neutrophils gathering in the alveolar space, alveolar capillary congestion and exudation, partial alveolar collapse, and alveolar space bleeding; these observations suggested significantly increased lung injury score of H/R rats than that of sham-operated rats (Figure 6B). At the same time, TUNEL staining found a more significant number of apoptotic cells in the lung tissue of H/R rats than in sham-operated rats (Figure 6C). These results demonstrated that the H/R rats had lung injury phenotypes, confirming the successful construction of our model. Moreover, compared with sham-operated rats, the H/R rats had significantly lower expression of miR-141-3p and higher SIRT1, Beclin-1 and LC3-II/LC3-I levels in lung tissue (Figure 6D, 6E). The results showed that the levels of miR-141-3p/SIRT1/Beclin-1 axis-related factors did change in the lung tissue of H/R rats, and the changing trend was similar to that of *in vitro* cells, along with increased autophagy in the lung tissue of H/R rats.

Subsequently, we treated H/R rats with mimic-NC + oe-NC, miR-141-3p mimic + oe-NC, miR-141-3p mimic + oe-SIRT1, or miR-141-3p mimic + oe-Belin-1. It was found that the symptoms of pulmonary edema were significantly alleviated in the H/R rats treated with miR-141-3p mimic, while further overexpression of SIRT1 or Beclin-1 could reverse the effects (Figure 6F). HE staining showed that overexpression of miR-141-3p could significantly reduce H/R-induced lung lesions and diminish the lung injury score; however, simultaneous overexpression of SIRT1 or Beclin-1 further aggravated lung tissue lesions and significantly increased lung injury score (Figure 6G). Meanwhile, TUNEL staining showed that the apoptotic cells in the lung tissue of the H/R rats treated with miR-141-3p mimic were significantly reduced, which could be counteracted by additional overexpression of SIRT1 or Beclin-1 (Figure 6H). Finally, the results of RT-qPCR and Western blot revealed that treatment with miR-141-3p mimics elevated miR-141-3p expression while diminishing the levels of SIRT1, Beclin-1, LC3-II/LC3-I in the H/R rats, the effects of which could be negated by additional overexpression of SIRT1 or Beclin-1 (Figure 6I, 6J).

Collectively, miR-141-3p could regulate cell autophagy and lung injury in H/R rats through the SIRT1/Beclin-1 axis.

## DISCUSSION

**Figure 7:**
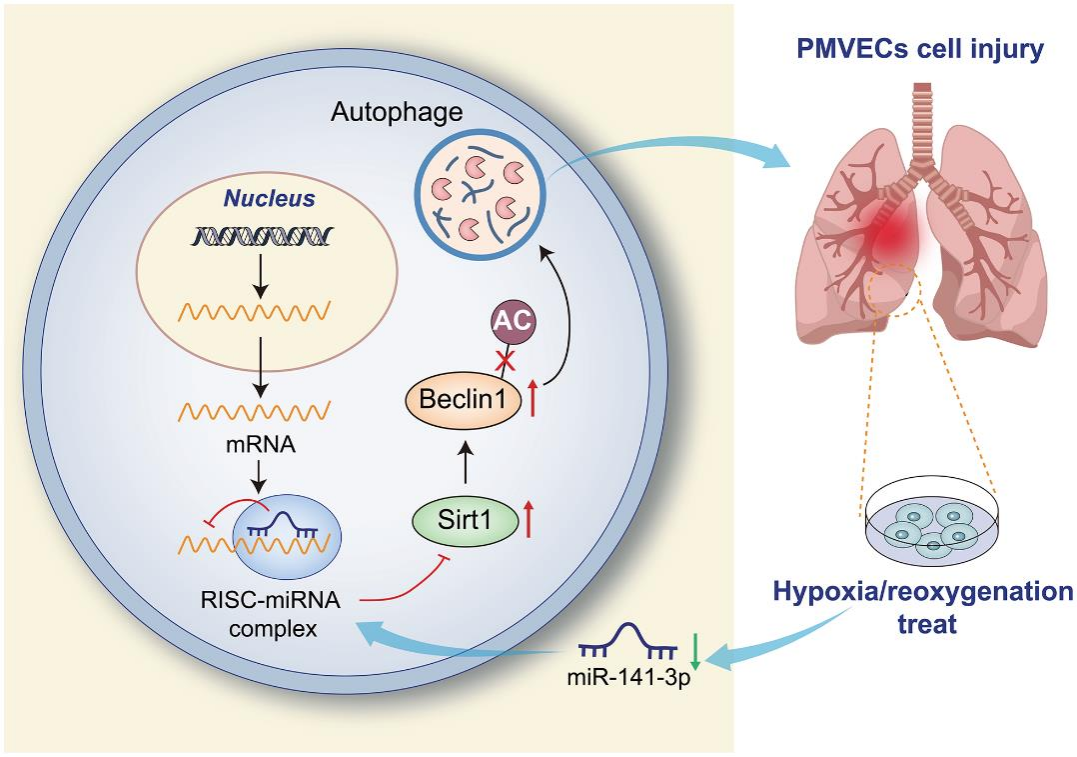
Overexpression of miR-141-3p downregulates Beclin-1 by targeting SIRT1 to inhibit autophagy and reduce H/R injury of PMVECs.

Pulmonary I/R injury is the leading cause of primary graft failure after lung transplantation, resulting in high morbidity and mortality [27]. Currently, no clinical therapies are specifically designed to prevent I/R injury [3]. PMVEC injury may lead to lung endothelial barrier impairment [28], and the destruction of PMVECs may affect the normal respiratory function of the lung, thereby reducing the cellular oxygen partial pressure of the lung tissue and inducing hypoxia response [29]. Therefore, the present study constructed an *in vitro* cell model of H/R using PMVECs, in combination with *in vivo* H/R rat models, aiming to explore the effects of miR-141-3p, SIRT1 and Beclin-1 in collaboration on H/R injury of PMVECs. Our results in *ex vivo* cultured PMVECs from rats showed that overexpression of miR-141-3p downregulates Beclin-1 by targeting SIRT1, thereby inhibiting autophagy and reducing H/R injury of PMVECs (Figure 7).

MiRNAs are essential mediators inhibiting gene expression by matching mRNAs from encoding genes [30]. Interest in exploring miRNAs’ role in specific diseases has increased recently. Numerous miRNAs are indicated to regulate autophagy in a variety of diseases, such as osteoarthritis [31], cardiac disease [32], and CNS inflammation [33]. Downregulated miR-141-3p by lncRNA-MALAT1 was unfolded to promote H9C2 cardiomyocyte pyroptosis induced by high glucose [34]. Additionally, H/R remarkably diminished miR-141-3p expression in H9C2 cardiomyocytes, and miR-141-3p overexpression could repress H/R-induced cardiomyocyte apoptosis [14].

Interestingly, our study showed that miR-141-3p was downregulated in PMVECs upon H/R, while overexpression of miR-141-3p suppressed autophagy, thereby attenuating the injury caused by H/R in lung PMVECs. Autophagy is an evolutionarily conserved mechanism that involves the degradation of unwanted or damaged cytoplasmic contents [35]. As autophagy plays a role as a trigger for apoptosis and necrosis [36], it is also considered relevant in the pathogenesis of many pulmonary diseases. Beclin-1 is a crucial gene for autophagy as it limits autophagy initiation, promotes autophagosome formation, and mediates autophagic activity [37]. Beclin-1 is upregulated upon H/R and contributes to autophagy-induced cell death where lysosome-associated membrane protein-2 synergizes with partial Beclin-1 knockdown to restore autophagosome processing after I/R [38]. The results of the present study suggested that H/R induced excessive autophagy, leading to cell death. One possible explanation is that excessive autophagy not only engulfs damaged organelles but also depletes typical organelles, leading to cell injury. In addition, excessive autophagosome production leads to insufficient binding of lysosomes with autophagosomes and incomplete degradation of autophagosomes, which damages the autophagy flux that further damages cells [39].

It is well known that miRNAs exert their biological functions by binding to the 3’-UTR of target genes. miRNAs are essential regulators of SIRT1. Several miRNAs, such as miR-34a and miR-181a, directly regulate SIRT1 expression through deacetylation [40]. MiR-141-3p was also reported to target and inhibit the expression of SIRT1 in Parkinson’s disease [16]. Additionally, miR-141-3p might share a negative correlation with SIRT1 protein expression [17]. In this study, through the TargetScan website, we predicted miR-141-3p may directly target the 3’-UTR of SIRT1 to inhibit its expression, which was further confirmed by luciferase reporter gene assay and RNA pull-down assay. SIRT1 is an NAD-dependent class III histone deacetylase and an essential regulator of autophagy [41]. In addition, forced SIRT1 expression could partially negate the protective role of miR-204 on H/R-induced apoptosis and autophagy [42]. SIRT1 expression was found to correlate positively with Beclin-1 [43], and SIRT1 can contribute to autophagy via deacetylation of Beclin-1 [44]. Our study revealed that overexpression of SIRT1 could increase autophagy and apoptosis in PMVECs by promoting the deacetylation of Beclin-1.

This study uncovers a novel molecular mechanism that regulates autophagy and cellular damage in pulmonary microvascular endothelial cells (PMVECs) during hypoxia/reoxygenation (H/R) treatment. Specifically, our investigation reveals that SIRT1, through its deacetylation activity, enhances the stability and expression of the Beclin-1 protein. Furthermore, H/R treatment downregulates miR-141-3p, resulting in increased SIRT1 expression levels, facilitating autophagy-induced cellular damage in PMVECs. Moreover, miR-141-3p inhibits Beclin-1 expression by targeting SIRT1, thereby weakening PMVECs’ response to H/R-induced injury. This study unveils a novel miR-141-3p/SIRT1/Beclin-1 signaling axis crucial for regulating autophagy and cellular damage in PMVECs during hypoxia/reoxygenation. Further exploration of this mechanism may provide valuable insights into the biological response of vascular endothelial cells under hypoxia/reoxygenation conditions and offer new targets and strategies for treating related diseases, such as cardiovascular disorders.

In conclusion, the present study demonstrated that H/R could induce autophagy by diminishing the expression of miR-141-3p, which upregulated SIRT1 and then promoted the deacetylation of Beclin-1, thus aggravating pulmonary H/R injury. This study confirmed that miR-141-3p played an essential role in pulmonary H/R injury, and the newly discovered miR-141-3p/SIRT1/Beclin-1 axis may provide some mechanistic insight into the injury. Nevertheless, whether serum miR-141-3p level can be used to evaluate successful lung transplantation and whether miRNA-based drugs can be applied in clinical lung transplantation remains unclear. Moreover, whether similar mechanisms exist in pulmonary alveolar epithelial cells warrants further exploration.

## MATERIALS AND METHODS

### Construction and grouping of H/R rat models

The male Sprague-Dawley (SD) rats used in this study (4-5 months) were purchased from Beijing Vital River Laboratory Animal Technology Co., Ltd. (Beijing, China). Rats were allowed free access to food and water under standard light and temperature conditions. The rat experiments began after the rats were acclimatized for one week. In brief [45], rats were placed in a sterile room where a continuous vacuum pump and an adjustable inlet valve controlled the air pressure. The ambient O_2_ pressure was reduced to the final atmospheric pressure of 225 mmHg to form 48 mm Hg O_2_ partial pressure (pO_2_), thus inducing hypoxia. The rats were exposed to hypoxia for 30 min, after which they were exposed to normal pressure and normal oxygen conditions for 48 h and sacrificed for subsequent experiments. The control rats (sham-operated rats) were kept in a sterile room under normal pressure and normal oxygen for 30 min and sacrificed for subsequent experiments.

The experimental rats were sham-operated as control rats or subjected to H/R modeling without further treatment or further treated with lentiviral vectors expressing mimic negative control (NC) + overexpression (oe)-NC, miR-141-3p mimic + oe-NC, miR-141-3p mimic + oe-SIRT1 or miR-141-3p mimic + oe-Becin-1 (n = 5). The lentiviral vectors expressing mimic NC, miR-141-3p mimic, oe-NC, oe-SIRT1 and oe-Becin-1 used in this study were designed and synthesized by GenePharma (Shanghai, China). One week before the construction of the H/R model, rats were anesthetized by intraperitoneal injection of ketamine thiazide (100 and 20 mg/kg, respectively), and then the above lentiviruses were injected into the proper lung tissue through the diaphragm (5 μL/2 × 10^8^/mL). After 3 minutes, the needle tip was pulled out, and the diaphragm was sutured [46].

### Determination of pulmonary edema

Pulmonary edema was estimated by comparing the lung wet/dry weight ratio. After the experiment, the lungs were taken out and weighed immediately to obtain the wet weight. The tissue was then dried in an oven at 90° C for 24 h, weighed again to obtain dry weight, and then calculated the lung wet/dry weight ratio.

### Hematoxylin and eosin (H&E) staining

Histological analysis of the lungs was performed by H&E staining. In short, at room temperature, the tissue sample was fixed in a 10% (v/v) formalin neutral buffer solution for 72 h and paraffin-embedded. Next, the tissue blocks were cut into sections (5 μM) and stained with hematoxylin for 7 min and then with eosin for 15 s at room temperature. Finally, these sections were observed under an optical microscope to detect morphological changes [47]. The lung injury score was performed by a committee-certified pathologist who did not know the treatment allocation of the sample. The lung injury score was divided into 4 categories based on the severity of alveolar congestion and hemorrhage, the infiltration of neutrophils in the alveolar or vascular wall, and the thickness of the alveolar wall/hyaline membrane formation. The severity of each category was scored from 0 (minimum) to 4 (maximum), and the total score was calculated by summing the scores in these categories. Four separate lung sections were graded in each animal to produce an average score [48].

### TUNEL tissue staining

According to the instructions of TUNEL staining kits (C1091, Beyotime, Shanghai, China), TUNEL staining was performed on paraffin-embedded lung sections. Apoptotic bodies were stained brown using deionized water instead of TdT enzyme as NC. At least 5 different visual fields were randomly selected to count the number of apoptotic cells and calculate the proportion based on the formula: proportion of apoptotic cells (%) = total number of apoptotic cells/total number of cells × 100%.

### Isolation and culture of rat PMVECs

A total of 6 Sprague-Dawley rats (male, 6-7 weeks, weighing 200-250 g, Beijing Vital River Laboratory Animal Technology Co., Ltd.) were reared in specific pathogen-free conditions. PMVECs of rats were isolated and cultured as described previously [20]. Rats were intraperitoneally anesthetized with 3% pentobarbital sodium (0.1 mL/100 g, The BSZH Scientific Inc., Beijing, China), and sterile PBS was perfused into the right ventricle. When the lung tissue became completely pale, the heart and lung tissues were quickly removed, and the pleural lung tissue was collected. After flushing, the tissues were cut into 1-3 mm^3^ blocks, inoculated evenly into a 25 cm^2^ culture flask, and incubated with 1.5 mL of DMEM complete medium containing 100 kU/L penicillin, 100 kU/L streptomycin, 90 kU/L heparin, 50 μg/L serum, vascular endothelial growth factor and fetal bovine serum (FBS). The medium was changed daily to remove blood cells. After 60 h of adhesion to the wall, the tissue blocks were carefully removed, and the medium was changed for subsequent culture. The medium was changed every 3 days until a monolayer of cells covered approximately 90% of the surface of the flask. Cells were treated with 0.25% trypsin and passaged. Cells at passage 3 were used for subsequent experiments.

### Establishment of cell models of H/R

Rat PMVECs under normoxic conditions were treated with DMSO, 3-MA, mimic NC or miR-141-3p mimic. PMVECs under H/R condition were treated with DMSO, 3-MA, Selisistat, SRT2104, sh-NC + oe-NC, sh-SIRT1 + oe-NC, sh-SIRT1 + oe-Beclin-1, mimic NC, miR-141-3p mimic, mimic NC + DMSO, miR-141-3p mimic + DMSO, miR-141-3p mimic + Rapamycin, mimic NC + oe-NC, miR-141-3p mimic + oe-NC, miR-141-3p mimic + oe-SIRT1, or miR-141-3p mimic + oe-Beclin-1.

Rat PMVECs were cultured in a conventional incubator (5% CO_2_, 95% air) under normoxic conditions. As for the H/R model, PMVECs were initially incubated for 0, 2, 4 and 8 h in a hypoxic incubator (5% CO_2_, 50% N_2_, 45% air) and transferred to a conventional incubator (5% CO_2_, 95% air) for 6 h [21]. Except for DMSO (10 mM, D2650, Sigma-Aldrich, St. Louis, MO, USA), Rapamycin (10 nmol/L, cat. S1039), SRT2104 (SIRT1-specific agonist also known as GS2245840; cat. #S7792), Selisistat (SIRT1-specific antagonist also known as EX527; cat. #S1541), and 3-MA (autophagy inhibitor 10 nM, S2767) were purchased from Selleck Chemicals (Houston, TX, USA). The PMVECs were respectively incubated with 3-MA (final concentration: 10 mM, SRT2104 (final concentration: 3 μM), Selisistat (final concentration: 10 μM) and Rapamycin (final concentration: 10 nmol/L) for 1 h before H/R [20]. All experiments were repeated three times independently.

### Cell transfection

Lentiviruses expressing short hairpin RNA to silence SIRT1 (sh-SIRT1), oe-SIRT1, or oe-Beclin-1, as well as miR-143 mimic, were constructed by GenePharma. The sequences are indicated in Supplementary Table 1. For lentivirus packaging, 293T cells were cultured in a DMEM complete medium containing 10% FBS and passaged every other day. When PMVECs were in a logarithmic growth phase, they were detached by trypsin and triturated to make 5 × 10^4^ cells/mL cell suspension and seeded into 6-well plates with 2 mL per well. The cells were cultured overnight at 37° C, virus (1 × 10^8^ TU/mL) was added into the cells for infection, and stably transduced cells were obtained for subsequent tests. miR-141-3p mimic (sense-5’-UAACACUGUCUGGUAAAGAUG-3’; antisense-5’-CAUCUUUACCAGACAGUGUUA-3’) and mimic NC (sense-5’-UCACAACCUCCUAGAAAGAGUAGA-3’; antisense-5’-UCUACUCUUUCUAGGAGGUUGUGA-3’) were transduced using Lipofectamine 3000 (Invitrogen, Carlsbad, CA, USA).

### Western blot

The cultured cells were detached by trypsin and lysed with enhanced radioimmunoprecipitation assay (RIPA) lysate containing protease inhibitors (Boster Biological Technology Co. Ltd., Wuhan, China). Lung tissue samples were dissolved and lysed in the enhanced RIPA lysate containing 2% protease inhibitor (Boster Biological Technology Co. Ltd.). Following protein quantification with bicinchoninic acid protein quantification kits (Boster) and protein separation with 10% sodium dodecyl sulfate-polyacrylamide gel electrophoresis (SDS-PAGE), the separated proteins were electrotransferred to polyvinylidene fluoride (PVDF) membranes. The membranes were blocked with 5% bovine serum albumin at room temperature for 2 h and incubated with diluted primary antibodies overnight at 4° C, including rabbit anti-LC3B (ab48394, 1:1000, Abcam, Cambridge, UK), rabbit anti-Beclin 1 (ab207612, 1:2000, Abcam), rabbit anti-Bcl-2 (ab196495, 1:1000, Abcam), rabbit anti-Bax (ab32503, 1:1000, Abcam), rabbit anti-cleaved caspase-3 (#9661, 1:1000, Cell Signaling Technology, Beverly, MA, USA), rabbit anti-caspase-3 (#9662, 1:1000, Cell Signaling Technology), rabbit anti-SIRT1 (ab189494, 1:1000, Abcam), rabbit anti-Ac-Lys (ab21623, 1:2500, Abcam), or rabbit anti-β-actin (ab179467, 1:5000, Abcam). Then, the membranes were further incubated with horseradish peroxidase (HRP)-labeled goat anti-rabbit immunoglobin G (IgG, ab97051, 1:10,000, Abcam) for 1 h at room temperature and added with enhanced chemiluminescence solution (Millipore, Billerica, MA, USA). Image Pro Plus 6.0 (Media Cybernetics, Rockville, MD, USA) was used to quantify the grayscale of bands, with β-actin as the internal reference.

### Reverse transcription-quantitative polymerase chain reaction (RT-qPCR)

Total RNA was extracted using the RNeasy Mini Kit (74004, Qiagen, Valencia, CA, USA) and reversely transcribed into cDNA using a reverse transcription kit (RR047A, Takara, Japan). miRNA was detected using miRNA First Strand cDNA Synthesis (Tailing Reaction) kits (B532451-0020, Shanghai Sangon Biotechnology Co. Ltd., Shanghai, China). RT-qPCR was performed using SYBR® Premix Ex TaqTM II (Perfect Real Time) kits (DRR081, Takara, Japan) and a qPCR instrument (ABI 7500, ABI, Foster City, CA, USA). Three duplicated wells were set for each sample. Sangon synthesized primers (Supplementary Table 2). The relative expression was calculated using the 2^-ΔΔCt^ formula with β-actin or U6 as an internal reference.

### Detection of autophagy

Cells were cultured on coverslips for 24 h, transfected with pmRFP-GFP-LC3 plasmid for 24 h using Lipofectamine™ 2000 (11668030, Invitrogen, Carlsbad, CA, USA, Lipofectamine 2000; Thermo Fisher Scientific), fixed with 4% paraformaldehyde, and imaged by confocal fluorescence microscopy (FV10i, Olympus, Tokyo, Japan). At least five fields were selected in each experiment to count the number of GFP and mRFP dots, and the number of DAPI-positive nuclei in the same field was counted. The number of dots/cells = the total number of dots / the total number of nuclei. The cell experiment was repeated 3 times independently.

### Cell counting kit-8 (CCK-8) assay

The cell suspension was seeded in a 96-well plate (100 μL/well) and was pre-incubated in a humidified incubator containing 5% CO_2_ at 37° C. The cell viability was detected by CCK-8 assay at 0, 24, 48 and 72 h after seeding. Next, the plate was added with CCK-8 solution (40203ES60, Shanghai Yeasen Biotechnology Co., Ltd., Shanghai, China) (10 μL/well) and placed in the incubator for 2 h. The optical density value at 450 nm was measured with a microplate reader (Molecular Devices, Sunnyvale, CA, USA).

### Transwell migration assay

According to the manufacturer’s protocol, the 24-well Transwell chamber (MAMIC8S10, Millipore) was used for cell migration assays. Briefly, 1 × 10^6^ transfected cells diluted with the serum-free medium were added to the apical chamber and 500 μL of medium containing 10% FBS was added to the basolateral chamber. The cells were incubated for 24 h at 37° C with 5% CO_2_ in humid conditions. After that, the Transwell chambers were removed, and the cells were fixed in 100% methanol for 30 min, stained with 0.5% crystalline violet (C3886, Sigma-Aldrich) for 20 min and observed under an inverted fluorescence microscope (TE2000, Nikon, Tokyo, Japan) with five random fields selected. The average number of migrating cells per group was counted.

### Flow cytometry

Cells in the logarithmic phase were seeded in 6-well plates (1 × 10^5^ cells/mL). Next, the cells were processed according to their grouping and three duplicated wells were set up for each group. Forty-eight hours later, the cells were centrifuged at 44 g for 5 min and then collected again. After the removal of the supernatant, approximately 50 μL of PBS remained. The cells were added with 500 μL of binding buffer, resuspended in each test tube, and incubated with 5 μL of AnnexinV-FITC and 5 μL of PI for 10 min at room temperature in the dark. Next, 5 min before detection by flow cytometry (Cube6, Partec, Munster, Germany), the cells were added with 5 μL PI in an ice bath avoided from light for 5 min. FITC was detected at 480 - 530 nm, and PI was detected at wavelengths greater than 575 nm. The ratio of apoptotic cells (%) = (number of late apoptotic cells in the upper right quadrant + number of early apoptotic cells in the lower right quadrant) / total number of cells × 100%.

### Immunoprecipitation (IP)

Cells in each group were lysed in lysis buffer [50 mM Tris-HCl (pH 7.4), 150 mM NaCl, 10% glycerol, 1 mM EDTA, 0.5 % NP-40 and protease inhibitor mixture] and centrifuged to move cell debris. After measurement of the protein concentration by BCA, the same amount of protein was taken from each group and made up to the same volume with cell lysate, followed by the incubation with 1 μg anti-Beclin 1 (ab207612, 1:30), IgG (ab172730, 1:100), and 15 μL protein A/G beads (B23201, Bimake, Houston, TX, USA) for 2 h. After washing the cell lysates three times, beads were collected by centrifugation, added with an equal volume of reducing sample buffer, and boiled at 100° C for 5 min. Samples were separated by SDS-PAGE and transferred to PVDF membranes (Millipore), followed by Western blot analysis.

### Analysis of protein stability based on cycloheximide

Cells were treated with 10 μM cycloheximide (HY-12320, MedChemExpress, Monmouth Junction, NJ, USA) for a period (0, 2, 4, 6, and 12 h) to block protein synthesis. Extracts were prepared and then analyzed for Beclin-1 protein expression by Western blot as described previously.

### Luciferase assay

It’s predicted by the TargetScan website that miR-141-3p has a binding site at 3’UTR of SIRT1. We inserted wild-type SIRT1 3’UTR gene fragment (SIRT1-WT) or binding site-mutant SIRT1 3’UTR gene fragment (SIRT1-MUT) into a pMIR-REPORT plasmid (AM5795, Ambion, USA). Luciferase reporter plasmids (SIRT1-WT and SIRT1-MUT) and miR-141-3p mimic or mimic NC and were co-transfected into HEK293T cells (Shanghai Beinuo Biotechnology, Shanghai, China). After 48 h of transfection, cells were collected, lysed, and detected by a luciferase detection kit (K801-200; Biovision, Mountain View, CA, USA). Luciferase activity was detected by a Glomax20/20 photometer (E5311, Promega, Madison, WI, USA).

### RNA binding protein immunoprecipitation (RIP)

RIP kits (0101, Guangzhou Geneseed Biotech Co., Ltd., Guangzhou, China) were used to detect the binding of miR-141-3p to SIRT1 3’UTR. Cells were lysed with an equal volume of RIPA lysate (P0013B, Beyotime) for 5 min, and centrifuged at 14000 rpm for 10 min at 4° C to take the supernatant. Part of the cell extract was taken as input, and part of it was incubated with rabbit anti-AGO2 (1:100, ab156870, Abcam) at room temperature for 30 mi, with rabbit anti-human IgG (1:100, ab109489, Abcam) as an NC. The sample was digested with proteinase K to extract RNA, which was used for subsequent RT-qPCR to detect miR-141-3p and SIRT1 expression.

### RNA pull-down

Cells were transfected with biotinylated NC and miR-141-3p probes (50 nM each, Shanghai Cloudseq Biotechnology Co., Ltd., Shanghai, China). After 48 h of transfection, cells were collected and incubated with cell lysate (Ambion, Austin, TX, USA) for 10 min. The lysate was incubated with M-280 streptavidin magnetic beads (LSKMAGT, Sigma-Aldrich) pre-coated with RNase-free and yeast tRNA (Sigma-Aldrich) at 4° C for 3 h. Finally, the expression of SIRT1 was determined by RT-qPCR.

### Statistical analysis

All data were measurement data, expressed as mean ± standard deviation, processed using SPSS 21.0 software (IBM Corp. Armonk, NY, USA). Differences between the two groups were compared using an unpaired *t*-test. Changes between multiple groups were compared using one-way analysis of variance (ANOVA) and the Tukey multiple comparison test. Two-way ANOVA was used for data comparison at different time points. *p* < 0.05 was considered a significant difference.

### Data availability statement

The datasets generated and analyzed during the current study are available from the corresponding author upon reasonable request.

## Supplementary Material

Supplementary Figures

Supplementary Tables
