# Supplementary material for: MicroRNA-141-3p reduces pulmonary hypoxia/reoxygenation injury through suppression of Beclin-1-dependent autophagy
Source: Aging (Albany NY). 2024 Jan 22;16(2):1352–73. doi: 10.18632/aging.205430 (PMC10866419; doi:10.18632/aging.205430)
Supplement: Supplementary Figures [file aging-16-205430-s001.pdf]

SUPPLEMENTARY FIGURES

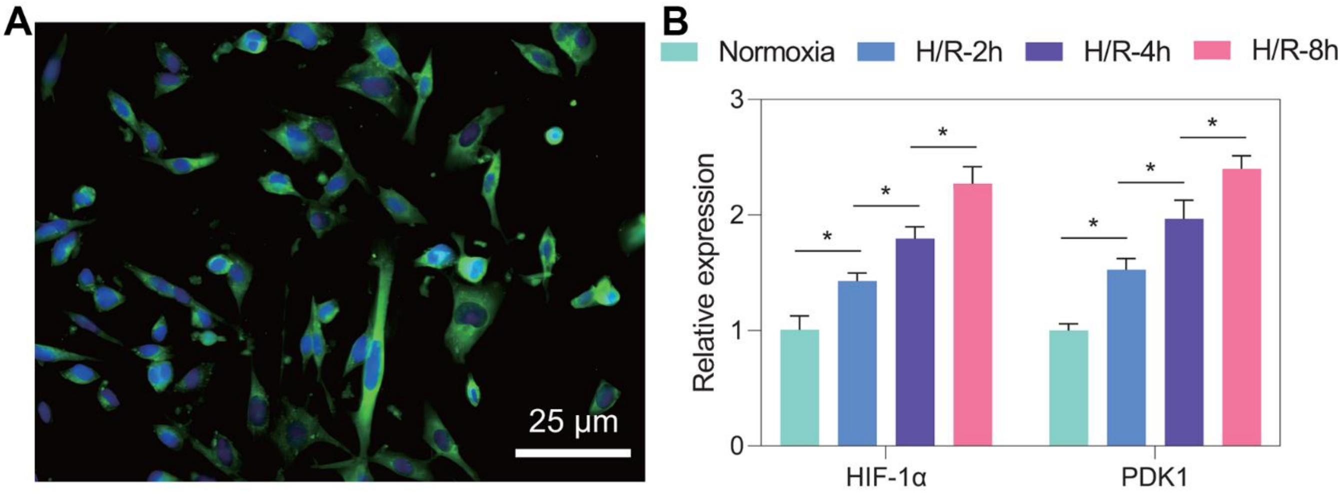

Supplementary Figure 1. Identification of PMVECs and hypoxia. (**A**) The expression of CD31 in PMVEC was detected by immunofluorescence assay (magnification, 200 ×; scale bar = 25 µm). (**B**) RT-qPCR to detect the expression of HIF-1α and PDK1 in PMVECs exposed to normoxia or hypoxia for a different period (2, 4, 8 h). Measurement data are expressed as mean ± standard deviation. Changes between multiple groups were compared using one-way ANOVA and Tukey's multiple comparison test. * $p < 0.05$. The cell experiments were repeated three times.

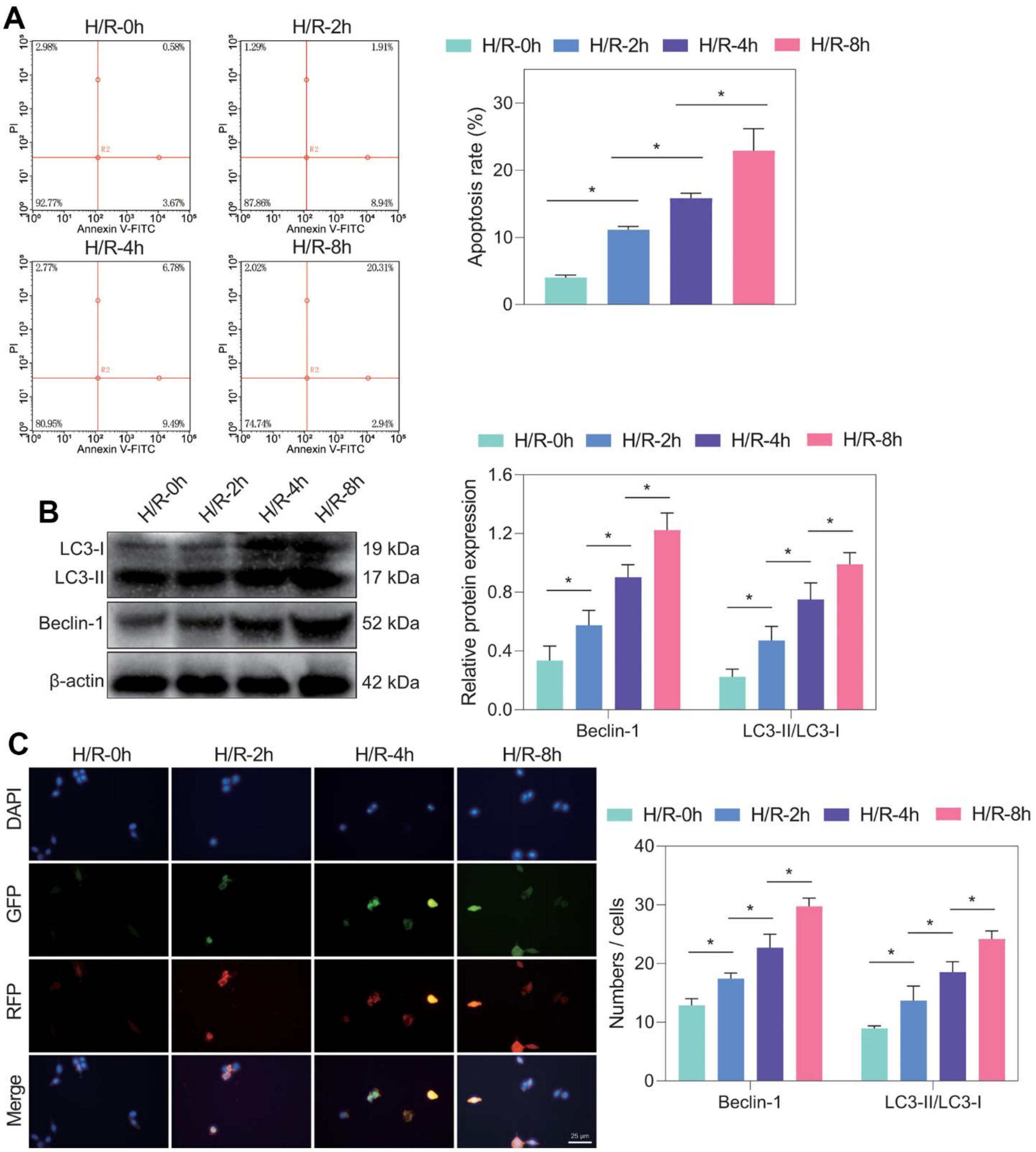

Supplementary Figure 2. Detection of autophagy and injury of H/R-treated PMVECs. (**A**) Flow cytometry to detect the apoptosis of PMVECs exposed to hypoxia for a different period (0, 2, 4, 8 h). (**B**) Western blot to detect the expression of Beclin-1, LC3-II/LC3-I in PMVECs exposed to hypoxia for different periods (0, 2, 4, 8 h). (**C**) mRFP-GFP-LC3 dual fluorescence system was used to trace autophagy formation in PMVECs exposed to hypoxia for different periods (0, 2, 4, 8 h).

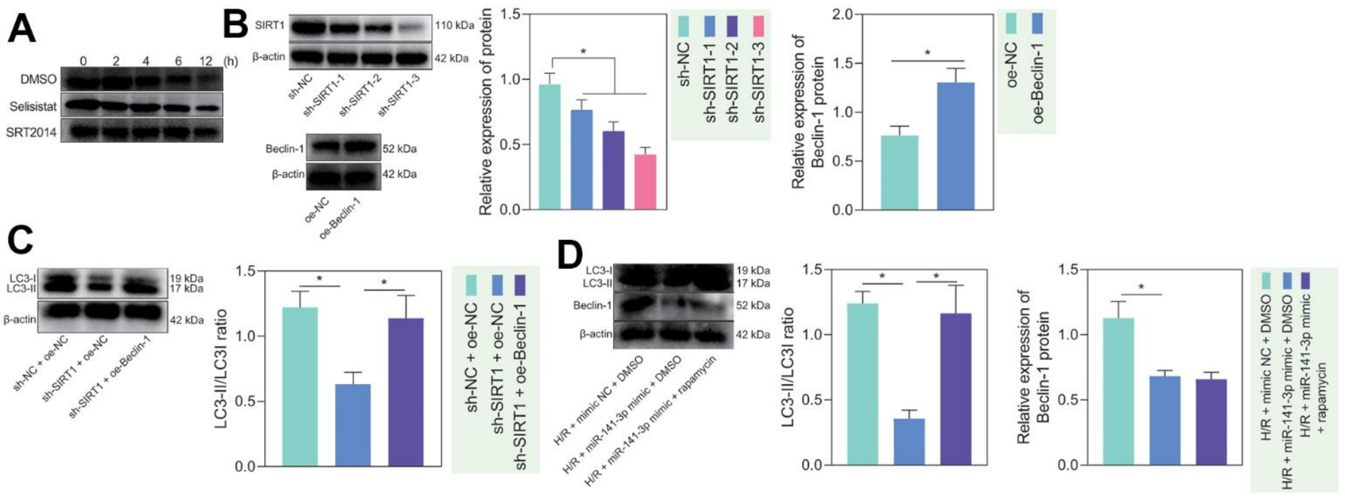

Supplementary Figure 3. (**A**) Stability analysis of Beclin-1 protein based on CHX treatment (Figure 2E). (**B**) Representative protein bands of SIRT1 and Beclin-1. (**C**) Representative protein bands of LC3-II/LC3-I in PMVECs treated with sh-SIRT1 or oe-Beclin-1. (**D**) Representative protein bands of LC3-II/LC3-I in PMVECs treated with H/R and miR-141-3p mimic. * $p < 0.05$. The cell experiments were repeated three times.
