# Supplementary material for: MicroRNA-141-3p reduces pulmonary hypoxia/reoxygenation injury through suppression of Beclin-1-dependent autophagy
Source: Aging (Albany NY). 2024 Jan 22;16(2):1352–73. doi: 10.18632/aging.205430 (PMC10866419; doi:10.18632/aging.205430)
Supplement: Supplementary Tables [file aging-16-205430-s002.pdf]

SUPPLEMENTARY TABLES

Supplementary Table 1. shRNA sequences.

shRNAs	Sequences
sh-SIRT1#1	5′-GGTTCCTTTGCAACAGCATCT-3′
sh-SIRT1#2	5′-GCAGATTAGTAAGCGTCTTGA-3′
sh-SIRT1#3	5′-TGTTTGGTCTACAAGTGTAGC-3′
sh-NC	5′-TTCTCCGAACGTGTCACGTTT-3′

Note: sh, short hairpin RNA; NC, negative control.

Supplementary Table 2. RT-qPCR primers.

	Forward primer	Reverse primer
SIRT1	5′-CTTGGAGCAGGTTGCAGGAAT-3′	5′-GGACACCGAGGAACTACCTGAT-3′
HIF-1α	5′-CTCAAAGTCGGACAGCCTCA -3′	5′-CCCTGCAGTAGGTTTCTGCT-3′
PDK1	5′-AAGAACTAAATGCGAAATCACC-3′	5′-TCAGCGGAACACCACCTC-3′
miR-141-3p	5′-TAACACTGUCTGGTAAAGATG-3′	Universal reverse primer
U6	5′-ACGCAAATTCGTGAAGCGTT-3′	5′-CTCGCTTCGGCAGCACA-3′
β-actin	5′-CTGGAACGGTGAAGGTGACA-3′	5′-AAGGGACTTCCTGTAACAATGCA-3′

Note: RT-qPCR, reverse transcription-quantitative polymerase chain reaction.
